# Susceptibility of Field-Collected *Nyssorhynchus darlingi* to *Plasmodium* spp. in Western Amazonian Brazil

**DOI:** 10.3390/genes12111693

**Published:** 2021-10-25

**Authors:** Diego Peres Alonso, Marcus Vinicius Niz Alvarez, Paulo Eduardo Martins Ribolla, Jan E. Conn, Tatiane Marques Porangaba de Oliveira, Maria Anice Mureb Sallum

**Affiliations:** 1 Biotechnology Institute and Bioscience Institute, Sao Paulo State University UNESP, Botucatu 18618-689, Brazil; marcus.alvarez@unesp.br (M.V.N.A.); p.ribolla@unesp.br (P.E.M.R.); 2 Departamento de Epidemiologia, Faculdade de Saúde Pública, Universidade de São Paulo, São Paulo 05508-060, Brazil; porangaba@usp.br (T.M.P.d.O.); masallum@usp.br (M.A.M.S.); 3 Wadsworth Center, New York State Department of Health, Albany, NY 12159, USA; jan.conn@health.ny.gov; 4 Department of Biomedical Sciences, School of Public Health, State University of New York, Albany, NY 12222, USA

**Keywords:** *Nyssorhynchus* *darlingi*, GWAS, NextRAD, cytochrome P450, chitinase

## Abstract

Mosquito susceptibility to *Plasmodium* spp. infection is of paramount importance for malaria occurrence and sustainable transmission. Therefore, understanding the genetic features underlying the mechanisms of susceptibility traits is pivotal to assessing malaria transmission dynamics in endemic areas. The aim of this study was to investigate the susceptibility of *Nyssorhynchus darlingi*—the dominant malaria vector in Brazil—to *Plasmodium* spp. using a reduced representation genome-sequencing protocol. The investigation was performed using a genome-wide association study (GWAS) to identify mosquito genes that are predicted to modulate the susceptibility of natural populations of the mosquito to *Plasmodium* infection. After applying the sequence alignment protocol, we generated the variant panel and filtered variants; leading to the detection of 202,837 SNPs in all specimens analyzed. The resulting panel was used to perform GWAS by comparing the pool of SNP variants present in *Ny.* *darlingi* infected with *Plasmodium* spp. with the pool obtained in field-collected mosquitoes with no evidence of infection by the parasite (all mosquitoes were tested separately using RT-PCR). The GWAS results for infection status showed two statistically significant variants adjacent to important genes that can be associated with susceptibility to *Plasmodium* infection: Cytochrome P450 (*cyp450*) and *chitinase.* This study provides relevant knowledge on malaria transmission dynamics by using a genomic approach to identify mosquito genes associated with susceptibility to *Plasmodium* infection in *Ny. darlingi* in western Amazonian Brazil.

## 1. Introduction

*Nyssorhynchus darlingi* (formerly *Anopheles (Nyssorhynchus) darlingi*) is the main malaria vector in the Neotropics throughout most of its distribution from Mexico to Argentina and, therefore, is the dominant malaria vector in the Brazilian Amazon River basin where transmission remains continuous and persistent, representing more than 99% of the country’s malaria burden [1,2]. In Brazil, 90% of malaria cases are caused by *Plasmodium vivax*, but in the Amazon Region, there is a proportion of cases caused by *Plasmodium falciparum*, which is associated with higher morbidity and mortality. The occurrence of *P. falciparum* malaria reflects pockets of heterogeneous malaria transmission with high infection rates in human and mosquito populations [3,4]. *Nyssorhynchus darlingi* possesses two major behaviors that are crucial in defining pathogen transmission to humans: anthropophily, i.e., the preference of a mosquito to take a blood meal on human hosts over other animals, depending on host availability and biomass; and endophagy—mosquito preference for blood-feeding indoors—although this adaptive species also feeds outdoors (exophagy) frequently [5,6,7,8,9]. In addition, *Ny. darlingi* is highly susceptible to human *Plasmodium* with a natural infection rate that varies between 0.24 and 3.96% in northern Brazil [10].

Mosquito susceptibility to *Plasmodium* infection is of paramount importance for malaria occurrence and sustainable transmission. Therefore, understanding the genetic features underlying *Ny. darlingi* susceptibility to infection is pivotal for investigating entomological components of malaria transmission dynamics in endemic areas. This knowledge can lead to the development of molecular tools for more precise vector surveillance and more effective interventions for malaria control.

Genetic structuring among natural populations of anopheline vectors at both the macro- and microgeographic scales leads to a high degree of nucleotide variation throughout the genome, a feature that ultimately influences both parasite transmission and the infection status of a mosquito vector [11,12,13,14]. Moreover, in the major African malaria vector *Anopheles gambiae*, several studies have used a gene candidate approach to identify factors that regulate mosquito-pathogen interactions, immunity, and parasite infection [13,15,16]. Despite a general lack of studies regarding genetic structuring and variation at the genomic scale in Neotropical malaria vectors, *Ny. darlingi* in the Amazon region has substantial variability in genetic structure and population divergence even at the microgeographic scale [12,17,18]. In addition, the genome of *Ny. darlingi* has a high level of nucleotide polymorphism with reported frequencies of up to 50 single nucleotide polymorphisms (SNPs) per 1000 base pairs in intronic and intergenic regions [19]. Based on these general concepts, genome-wide association studies can be performed to detect SNPs markers linked to genes predicted to modulate *Plasmodium* infection susceptibility in natural populations of *Ny. darlingi*.

The development of new strategies and advanced technologies related to whole-genome sequencing (WGS) has resulted in dramatic reductions in sequencing cost and effort. Nevertheless, studies that require sequencing a large number of samples remain costly and thus are not currently feasible. Reduced representation of the genome-sequencing protocols such as Nextera-tagmented, reductively amplified DNA (NextRAD) and derivative approaches that generate SNP datasets are powerful technologies for the assessment of a large number of SNPs on a genome-wide scale in anophelines with considerably lower cost that can represent up to a 40-fold decrease in economical effort for library construction and sequencing [12,17,19]. Here, we report a genome-wide association study using NextRAD-derived SNP markers to identify mosquito genes predicted to modulate the susceptibility of natural populations of *Ny. darlingi* to *Plasmodium* infection in the western Amazon.

## 2. Materials and Methods

### 2.1. Study Sites and Adult Mosquito Collection

**Figure 1:**
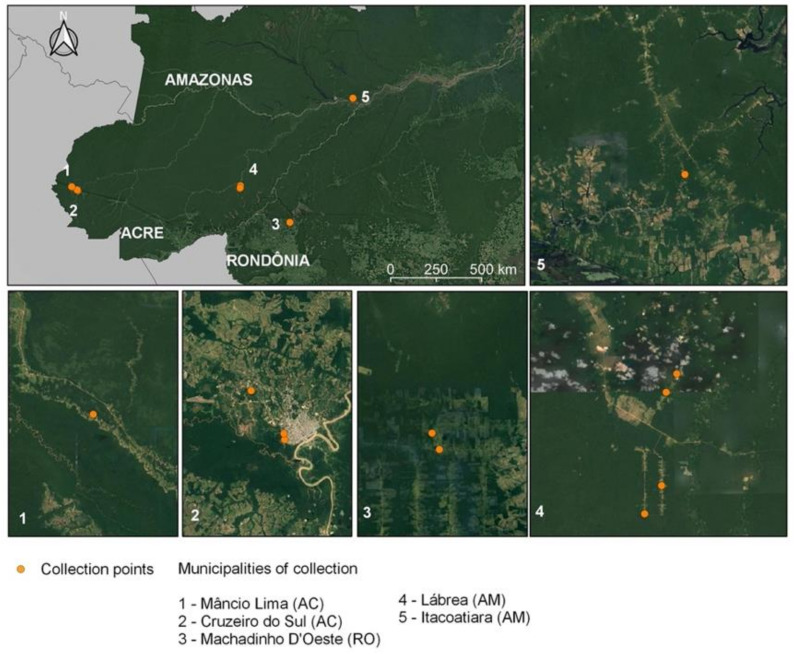
**Collection region of Brazilian Amazon**. Map depicting Acre, Amazonas and Rondônia states with the 11 collection sites distributed in the municipalities of Mâncio Lima, Cruzeiro do Sul, Machadinho D’Oeste, Lábrea and Itacoatiara.

Females of the subfamily Anophelinae were collected from a total of 11 houses distributed in five municipalities in the Brazilian Amazon states of Acre, Amazonas, and Rondônia. The municipalities of Cruzeiro do Sul and Mâncio Lima are in the region of the Juruá River basin, western Acre state. Machadinho D’Oeste is situated in the Machadinho River basin along highway BR-364 in Rondônia state. For the Amazonas municipalities, Lábrea is alongside the Boa Água River—a tributary of the Purus River west of the BR-230 Brazilian Trans-Amazonian highway. Itacotiatiara is in the metropolitan region of Manaus along the Amazon River (Figure 1).

Female collections were conducted from April 2015 to October 2015, except the collection in Itacoatiara municipality, Amazonas state, which was in November 2016. Field collections were carried out during the wet–dry transition as well as in the dry season (Table S1). Mosquitoes were collected outdoors in the peridomestic environment within ~ 5 m of each of 11 houses. Human landing catch and barrier screen interception collections were performed for one night in each house from 18:00 to midnight [10], except in Cruzeiro do Sul and Mancio Lima, Acre state, where collections were performed from 18:00 to 06:00 am (Table S1). Every hour, female mosquitoes were euthanized with ethyl acetate (C_4_H_8_O_2_) vapors in the field and stored immediately in silica gel separated by date, location, house, and hour of collection. Specimens were morphologically identified to species level, and *Ny. darlingi* were labeled and stored individually with silica gel at room temperature for subsequent analysis. Regarding study design, *Ny*. *darlingi* female samples were paired as infected and uninfected by locality and hour of collection whenever it was possible. Afterwards, we randomly selected uninfected individuals of the same localities totaling 36 infected and 29 non-infected females.

### 2.2. Mosquito Processing and NextRAD

Genomic DNA was extracted from the thorax/head of *Ny. darlingi* adult females using Qiagen DNeasy Blood & Tissue Kit (Hilden, Germany). *Nyssorhynchus darlingi* DNA samples were tested for *Plasmodium* spp. infection following [20,21], except for the DNA pools of three individuals containing equal amounts of gDNA. If a pool was positive for real-time PCR amplification, all three individuals that originally composed the pool were tested separately [10], using both *Plasmodium* spp. positive and several negative controls in each run. In addition, all individuals employed in the study that tested negative for *Plasmodium* were individually double-checked by RT-PCR. Each PCR reaction contained 1× PerfeCTa qPCR ToughMix, Uracil *N*-glycosylase (UNG), ROX (Quanta Biosciences, USA), 0.3 μM of each primer, ultrapure water, and 2 μL genomic DNA for a total volume of 20 µL. Cycling conditions were as follows: 5 min UNG-activation at 45 °C and a denaturation step for 10 min at 95 °C followed by 50 cycles of 95 °C denaturation for 15 s and 60 C annealing/elongation for 1 min. For the NextRAD protocol, DNA samples of the thorax/head of females were sent to SNPsaurus LLC (Eugene, OR) for library preparation and NGS sequencing. To construct DNA libraries, genomic DNA (~10 ng) was first fragmented with Nextera transposase (Illumina, San Diego, CA), which also ligated short adapter sequences to the ends of the fragments. DNA fragments were then amplified with two primers matching adaptor sequences with one of the primers extending an additional nine nucleotides (GTGTAGAGC) as the selective sequence at the 3′ end. Thus, only fragments that could be hybridized to the selective sequence were efficiently amplified. The libraries were sequenced on an Illumina HiSeq2000 with 1 × 150 bp configuration to generate ~50 X coverage.

### 2.3. Variant Calling

The *Ny. darlingi* reference genome is available in the VectorBase database. Version AdarC3 [22] was used to align raw NextRAD reads. Alignments were performed with Burrows-Wheeler Aligner (BWA) software [23], and variant calling used SamTools and BcfTools [24]. A genotypes panel was generated in VCF 4.2 format. VCF quality control was applied with VCFtools [25]. Genotypes were removed if DP < 5 and GQ < 20, and SNPs were filtered for MAF > 0.1 and MD < 0.8 within case and control groups.

### 2.4. Genome-Wide Association Study (GWAS)

The genetic association analysis was performed using Fisher’s Exact test with PLINK 1.9 package tool [26]. Case and control categories were the *Plasmodium*-infected (regardless of their species) and *Plasmodium* non-infected mosquitoes, respectively. The false discovery rate (FDR) multiple test correction method was applied to control for false positives assuming statistical significance with a corrected *p*-value < 0.05. Manhattan Plot images were generated using Rstudio for R language [27]. Adjacent protein-coding genes up to 80 kb from FDR-significant SNPs were investigated using AdarC3 from the annotated *Ny. darlingi* genome available in the gff3 format in VectorBase.

### 2.5. Genetic Structure Testing

In order to evaluate any possible bias produced by a significant genetic structure signature on the GWAS analysis, we conducted a *Fst* pairwise analysis using Arlequin 3.5 [28] for the five localities and also for *Plasmodium*-infected (regardless of their species) and *Plasmodium* non-infected mosquitoes.

### 2.6. Protein Sequence Comparisons

Following SNP identification adjacent to protein-coding genes by GWAS analysis, we performed a sequence comparison of these gene products with all correlated proteins found in *An. gambiae*, since there are numerous studies regarding gene functions and characterization for this species. We used Blastp to search all available proteins sequences in GenBank, restricted to taxid 7165 that represents *An. gambiae* and taxid 180, 454 that represents *An. gambiae* str. PEST using non-redundant protein sequences as the database.

## 3. Results

Following species identification, all *Ny. darlingi* females selected for the study (*n* = 65) were submitted to DNA extraction and RT-PCR tested for *Plasmodium* spp. infection. There were 36 specimens infected: 26 with *P*. *vivax* and 10 with *P. falciparum*. Additionally, 29 specimens from the same collection sites were PCR-negative for *Plasmodium* and used for the genomic analysis (Table 1).

From a total of 48 Gigabases of sequence data, ~5 million 150 bp reads were obtained from each *Ny. darlingi* female NextRAD library.

**Figure 2:**
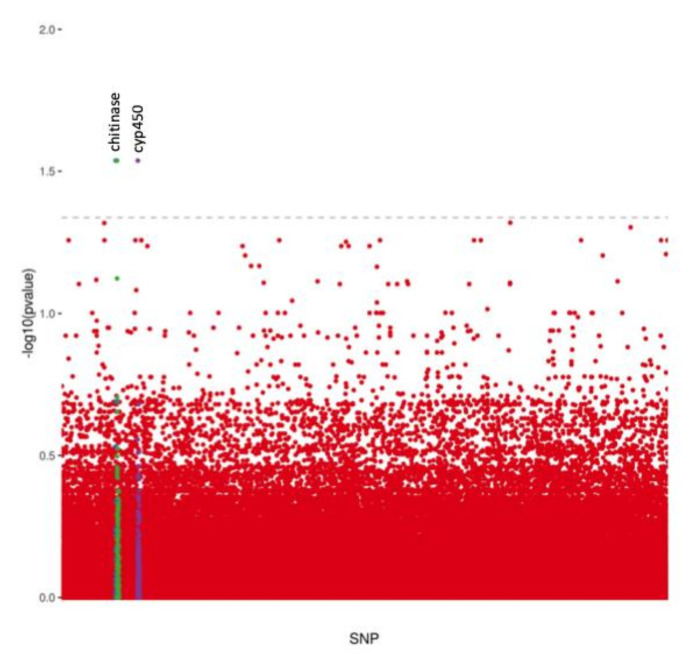
**GWAS analysis results for *Plasmodium* infection**. Horizontal dashed line represents the significance threshold of 0.05 for the FDR-corrected *p*-value. Highlighted colors represent the scaffolds containing significantly associated SNPs.

After applying the sequence alignment protocol, generating the variant panel, and filtering variants, 202,837 SNPs were obtained and present in all individuals analyzed. These SNPs were used for a genome-wide association study comparing the pool of SNP variants present in *Ny. darlingi* infected with *Plasmodium* (cases) with the pool obtained from mosquitoes that did not show any evidence of infection by the parasite (controls). Fisher’s exact test was performed to compare *Plasmodium*-infected and *Plasmodium* non-infected mosquitoes. The results of the analyses disclosed two different scaffolds with significantly associated SNPs separated less than 80 Kb from *cyp450* (ADAC009195) and *chitinase* (ADAC002977) genes (Figure 2). The results of Fisher’s allelic association test with *post hoc* FDR showed that the frequencies of the SNPs of interest and the genes adjacent to the SNPs were statistically related to *Plasmodium* infection (Table 2). To further investigate the association of SNPs with infection status, we repeated the GWAS analysis using only *P. vivax* infected mosquitoes as the case group. Likewise, a GWAS analysis was also undertaken using, this time, only *P. falciparum* as the case group. Interestingly, for the *P. vivax* analysis, the SNP associated with *cyp450* was retained while the *chitinase* SNP was no longer present. Conversely, in the *P. falciparum* analysis, the results obtained did not show any SNP associated with the infection status (Figure S1). This result indicates that despite not effectively contributing to the observed correlation with the SNP linked to *cyp450*, *Ny. darlingi* infected with *P. falciparum*, to some extent, took part in the disclosure of the *chitinase*-associated SNP. The fact that there was no such observed association in the analysis that used only *P. falciparum*-infected mosquitoes is probably due to the low number of samples analyzed.

Importantly, no significant genetic structure was detected for localities or group assignment (case/control) by *Fst* estimation (Table S2). In fact, significant *Fst* pairwise values were detected for Lábrea versus Machadinho D’Oeste populations, and for Cruzeiro do Sul versus Itacoatiara populations. However, the *Fst* value found is extremely low for the Lábrea versus Machadinho D’Oeste comparison. Because the Itacoatiara population is composed of only two females, the estimate of a real *Fst* pairwise difference was irrelevant.

*Nyssorhynchus darlingi* versus *An. gambiae* protein sequence comparisons for *cyp450* and *chitinase,* returned, respectively, the following well-characterized genes for the top five blastp hits: *CYP6Z2*, *CYP6Z3*, and *AgCht6*, *AgCht8*, and *AgCht9* (Table S3).

## 4. Discussion

The results of the genome-wide association analyses verified the genetic association of *Plasmodium* infection and showed two statistically significant variants (Figure 1). The investigation of genes adjacent to the significantly associated *loci* identified two important genes that might play a major role in *Ny. darlingi* infection with *Plasmodium*: cytochrome P450 (*cyp450*) and chitinase (*chitinase*). The cytochrome P450 gene superfamily is composed of more than 70 families consisting of a major metabolic system responsible for the regulation of endogenous compounds such as hormones, fatty acids, and steroids. In addition, P450 is linked to the catabolism and anabolism of xenobiotics such as drugs and pesticides in distinct groups of organisms such as plants, mammals, birds, and insects [29]. The cytochrome P450 genes are associated with insecticide resistance through increased metabolism of insecticides in *Culex quinquefasciatus*, *Aedes aegypti*, and *Anopheles funestus* [30,31,32,33]. In *An. gambiae*, five genes of the superfamily were demonstrated to be linked to pyrethroid resistance: *CYP6Z1*, *CYP325A3, CYP6Z2*, and *CYP6M2* [34,35]. In *Ny. darlingi*, *cyp450* led to 73% protein sequence similarity with *CYP6Z2* of *An. gambiae.* Interestingly, in this latter vector species, *CYP6Z2* is expressed at high levels in the larvae but is also expressed in adults suggesting that it is potentially involved in pyrethroid resistance in the adult stage. Moreover, the *CYP6Z2* gene product is localized in Malpighian tubules and in the midgut of insecticide-resistant mosquitoes: These are the first tissues of the mosquito to interact with *Plasmodium* after a blood meal [36,37].

Considering the biological function of the *cyp450* gene, it is clear that the presence of an adjacent SNP can be linked to the use of pyrethroid insecticides in indoor residual spraying (IRS) and long-lasting insecticidal nets (LLINs) inside dwellings as vector control interventions. In this scenario, polymorphisms in individuals that are more resistant to insecticides might lead to a greater chance of survival in an environment with high exposure to insecticide. In *Plasmodium* infection, it is tempting to propose a hypothesis wherein the parasite can indirectly exploit the phenotypic behavioral adaptation of anophelines to improve transmission to humans. Considering that female *Ny. darlingi* can feed on blood both indoors and outdoors [7,38], they must be able to survive inside houses that are treated with IRS. Consequently, the number of infective bites may increase in a situation where females are tolerant to residual insecticide. This capacity allows the anopheline female to have a high contact rate with both infectious and susceptible human hosts because of mosquito feeding behavior and tolerance to insecticide.

Chitinases are hydrolytic enzymes that break down glycosidic bonds in chitin. They are found in several organisms, including vertebrates, microorganisms, and plants [39]. In insects, chitin is associated with proteins to form the cuticular exoskeleton and peritrophic matrix (PM) in the midgut lumen. Insect chitinases have been suggested to have multiple functions, including defense, digestion, and molting [40,41,42]. The chitinase gene (*chitinase*) is essential for the correct metabolism and processing of chitin, and it is ubiquitous in anophelines with more than 20 variants already described [43]. The *chitinase* enzyme plays a key role in the degradation and stabilization of the PM in anophelines and insects in general [44]. The *Ny. darlingi* chitinase gene identified here showed 43% protein sequence similarity with the *AgCht8* of *An. gambiae.* This gene is mainly expressed in the pupal and adult stages [43]; this enzyme is the fifth most abundant protein in *An. gambiae* PM [44].

The first step for the invasion of the parasite in the mosquito vector is to successfully invade the PM that, at the time of invasion separates the food bolus from the midgut epithelium of the mosquito. This pivotal degradation process is normally carried out by a chitinase secreted by the parasite. Interestingly, chitinase inhibitors and anti-chitinase antibodies are known to reduce parasite transmission from the vertebrate host to the mosquito, thus suggesting that the peritrophic matrix chitin is a critical feature related to *Plasmodium* penetration of the midgut [45,46,47]. Considering the major roles of chitinases in the crucial processes of the *Plasmodium* invasion, it is reasonable to presume that genetic variants of mosquito chitinases can be responsible for the integrity and variable thicknesses of the PM. Interactions between anopheline chitinases and *Plasmodium* chitinases might effectively interfere with the process of invasion of the midgut epithelium with a substantial impact on the success of *Plasmodium* infection.

Taken together, these data suggest two classes of metabolically important genes—*cyp450* and *chitinase*—are likely to be related to *Plasmodium* infection in the Brazilian Amazon. The results of the allelic association test performed in this study showed that the frequencies of the SNP variants for *cyp450* and *chitinase* (Table 2) were significantly higher in *Ny. darlingi* infected with *Plasmodium.* This may partially explain the genetic background related to *Plasmodium* susceptibility in *Ny. darlingi*.

There is a scarcity of studies on the association of genetic polymorphisms with natural resistance to *Plasmodium* infection in anophelines, with only a couple of publications in recent years addressing this complex host-parasite interaction [48,49]. It is noteworthy that these studies disclosed SNPs only in *loci* responsible for coding immune genes of *An. gambiae*, such as *TOLL11*, *TOLL6*, *AgMDL1*, and *CEC1*. Moreover, all experiments were carried out in lab-reared mosquitoes with artificial *Plasmodium* infection approaches, which, in turn, lead to extreme phenotypes with unrealistic loads of parasites, ultimately representing a scenario extremely far from natural. The major drawback of such studies is that they are unable to evaluate genetic traits influencing physiological or behavioral resistance to *Plasmodium* infection, where genes related to blood-feeding behavior, resting behavior, host-seeking, and differential longevity might play a major role in the infection process. In fact, in a study conducted with several field-collected *An. gambiae* populations in Africa, the authors reported that two La inversion alleles are associated with natural malaria infection levels and in wild-captured vectors. Besides that, mosquitoes carrying the more susceptible allele (2L+^a^) are also behaviorally less likely to be found inside houses [50].

## 5. Conclusions

To our knowledge, the study presented here, with all its intrinsic limitations (e.g., limited sample size, low marker density, low overall genome linkage disequilibrium) is the first to use field-collected *Ny. darlingi* to assess natural susceptibility to *Plasmodium* infection. Further studies are needed to better characterize the functions of *cyp450* and *chitinase* genes as key factors that regulate mosquito-parasite interactions. Finally, reduced representation in the genome-sequencing protocols associated with genome-wide genetic association analysis may enhance the development of molecular tools to better understand the genetic traits of *Ny. darlingi* to improve vector surveillance and malaria control.

## Figures and Tables

**Table 1:** List of adult *Ny. darlingi* females used in the study by locality and infection status.

Municipality	State	*P. vivax*	*P. falciparum*	Negative	Total
Cruzeiro do Sul	Acre	17	-	17	34
Mâncio Lima	Acre	-	1	1	2
Lábrea	Amazonas	3	9	6	18
Machadinho D’Oeste	Rondônia	5	-	4	9
Itacoatiara	Amazonas	1	-	1	2
Total	26	26	10	29	65

**Table 2:** List of statistically significant markers (*p_FDR_* < 0.05) and adjacent genes in GWAS for *Plasmodium* infection. Adjacent genes located in a maximum range of 80 kb are described.

Scaffold	Position	Ref	Alt	Adjacent Genes	Distance to Adjacent Gene (bp)	*p_FDR_*
ADMH02000716	10,949	G	C	*cyp450*	56	0.029
ADMH02002098	10,391	T	A	*Chitinase*	71,655	0.029

Ref: reference allele. Alt: alternative allele. *p_FDR_*: false discovery rate corrected *p*-value.

## Data Availability

The datasets generated and analyzed during the current study have been deposited at NCBI Bi-oProject database under the accession number PRJNA773638.

## Supplementary Materials

The following are available online at https://www.mdpi.com/article/10.3390/genes12111693/s1, Table S1: Description of all specimens collected and used in the study. Table S2: Pairwise *Fst* values for population and case/controls comparisons. Table S3: Top 5 blastp results for *chitinase* and *cyp450* gene products. Figure S1: Fisher test results for *P. vivax* vs. non-infected and *P. falciparum* vs. non-infected comparisons.
