# Supplementary material for: Susceptibility of Field-Collected Nyssorhynchus darlingi to Plasmodium spp. in Western Amazonian Brazil
Source: Genes (Basel). 2021 Oct 25;12(11):1693. doi: 10.3390/genes12111693 (PMC8623036; doi:10.3390/genes12111693)
Supplement: Supplementary file 1 [file genes-12-01693-s001.zip › Figure S1.pdf]

Figure S1. Fisher test results. (a) Case (*P. vivax*) vs Control (Non-infected) and (b) Case (*P. falciparum*) vs Control (Non-infected)

a)

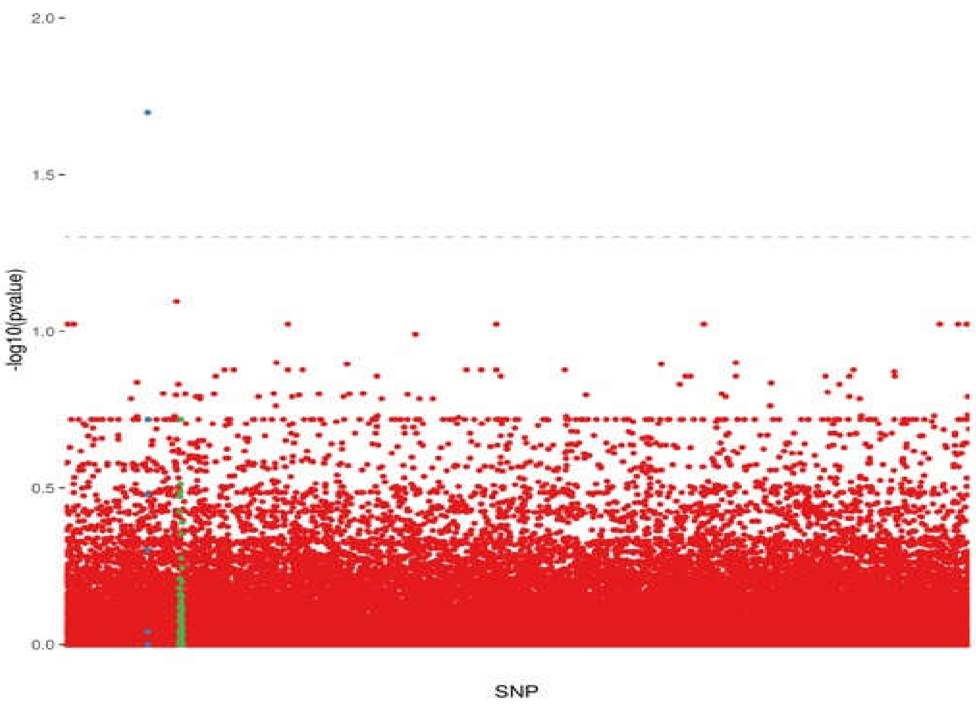

SNP	FDR_BH
ADMH02000716:10949:G:C	0.02003
scaffold_1270:26864:T:C	0.08033
scaffold_97:193554:G:C	0.09482
scaffold_102:180948:T:C	0.09482
scaffold_291:76943:C:A	0.09482
scaffold_1010:36619:GC:G	0.09482
scaffold_940:8710:G:A	0.09482
scaffold_534:51659:T:A	0.09482
scaffold_162:91422:C:T	0.09482
scaffold_99:13893:T:C	0.09482

b)

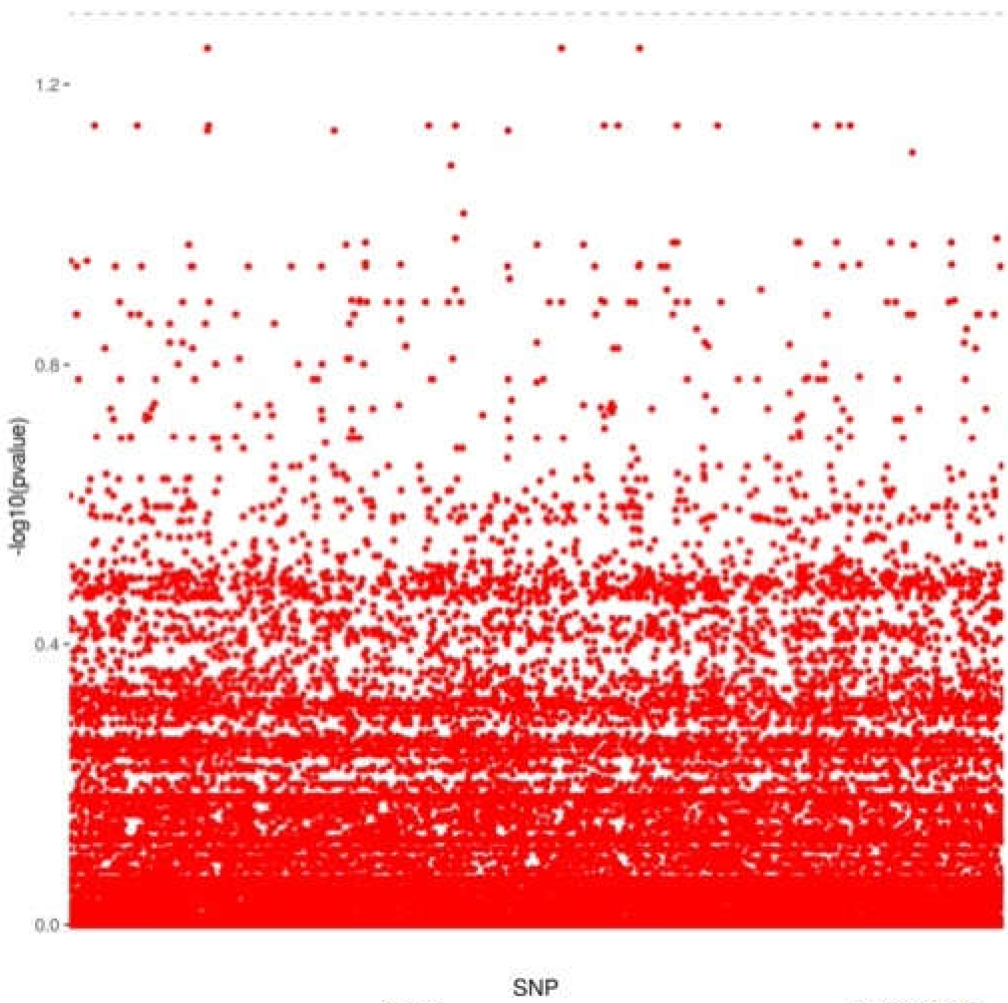

SNP	FDR_BH
scaffold_430:54055:G:C	0.05595
scaffold_13:273450:C:A	0.05595
scaffold_13:273475:GCGACTTCTGCAGCGACGAC:GCGAC	0.05595
scaffold_345:89182:A:T	0.05595
scaffold_522:18575:A:G	0.07221
scaffold_105:212267:G:A	0.07221
scaffold_650:38916:C:G	0.07221
scaffold_650:38918:G:C	0.07221
scaffold_691:37040:GTTT:GTT	0.07221
scaffold_222:92892:C:G	0.07221
