# Supplementary material for: Susceptibility of Field-Collected Nyssorhynchus darlingi to Plasmodium spp. in Western Amazonian Brazil
Source: Genes (Basel). 2021 Oct 25;12(11):1693. doi: 10.3390/genes12111693 (PMC8623036; doi:10.3390/genes12111693)
Supplement: Supplementary file 1 [file genes-12-01693-s001.zip › Table S2.pdf]

Table S2. Pairwise *Fst* values for population comparisons (a) and case/controls comparisons (b).

a)

	Cruzeiro do Sul	Mâncio Lima	Lábrea	Machadinho D'Oeste	Itacoatiara
Cruzeiro do Sul	*				
Mâncio Lima	0.065	*			
Lábrea	0.000	0.055	*		
Machadinho D'Oeste	0.007	0.055	0.015*	*	
Itacoatiara	0.108*	0.114	0.007	0.017	*

*$P < 0.001$

b)

	P. vixax	*P. falciparum*	Non-infected
P. vixax	*		
P. falciparum	0.000	*	
Non-infected	0.000	0.003	*

*$P < 0.001$
