# Supplementary material for: Susceptibility of Field-Collected Nyssorhynchus darlingi to Plasmodium spp. in Western Amazonian Brazil
Source: Genes (Basel). 2021 Oct 25;12(11):1693. doi: 10.3390/genes12111693 (PMC8623036; doi:10.3390/genes12111693)
Supplement: Supplementary file 1 [file genes-12-01693-s001.zip › table S3.pdf]

Table S3 – Top 5 blasp results for (a) chitinase and (b) cyp450.

a)

Description	Query Cover	E value	Per. Ident	Accession	VectorBase Gene identity
AGAP011033-PA [Anopheles gambiae str. PEST]	76%	0.0	65.01%	XP_307732.4	AgCht8
AGAP009022-PA [Anopheles gambiae str. PEST]	81%	2,00E-146	32.15%	XP_001238192.2	AgCht9
AGAP006898-PA [Anopheles gambiae str. PEST]	79%	2,00E-139	31.44%	XP_308858.4	AgCht6
chitinase [Anopheles gambiae]	82%	2,00E-115	42.61%	AAB87764.1	-
AGAP006414-PA [Anopheles gambiae str. PEST]	82%	1,00E-114	43.23%	XP_316448.2	AgCht8

b)

Description	Query Cover	E value	Per. Ident	Accession	VectorBase Gene identity
cytochrome P450 CYP6Z2 [Anopheles gambiae]	96%	0.0	73.32%	ABV80276.1	-
AGAP008218-PA [Anopheles gambiae str. PEST]	96%	0.0	73.32%	XP_317252.1	CYP6Z2
AGAP008217-PA [Anopheles gambiae str. PEST]	96%	0.0	72.30%	XP_555249.1	CYP6Z3
cytochrome P450 CYP6Z2 protein [Anopheles gambiae]	96%	0.0	73.12%	AAL96667.1	-
cytochrome P450 [Anopheles gambiae]	96%	0.0	72.10%	AAO24698.1	-
